# Development and Clinical Application of a New Open‐Powered Nail Anterior Cervical Plate System

**DOI:** 10.1111/os.12621

**Published:** 2020-02-19

**Authors:** Xiao‐feng Zhao, Yi‐bo Zhao, Xiang‐dong Lu, De‐tai Qi, Xu Yang, Wen‐xuan Wang, Xiao‐nan Wang, Run‐tian Zhou, Yuan‐zhang Jin, Bin Zhao

**Affiliations:** ^1^ Department of Orthopaedics The Second Hospital, Shanxi Medical University Taiyuan China

**Keywords:** Anterior cervical spine plate, Clinical efficacy, New type

## Abstract

**Objective:**

To observe and evaluate the clinical curative effect of a new type of open‐powered cervical spine system developed for anterior cervical surgery.

**Methods:**

A retrospective analysis was performed in our hospital in 2015–2017 of 329 orthopaedic patients treated with cervical anterior decompression, cage or titanium mesh graft fusion, new open‐powered nail plate or traditional cervical anterior screw plate. A total of 154 (control group) and 175 (observation group) cases were fixed with conventional cervical‐ and new open‐powered nail plates, respectively. Postoperative follow‐up was performed. Cervical stability, internal fixation position, and bone graft fusion were evaluated by imaging. Operative time, intraoperative blood loss, cervical Cobb angle, pain visual analogue scale (VAS) score, and Japanese orthopaedic association (JOA) score were compared between the groups. JOA scoring (spinal cord function) and neurological function improvement rate (IR) were used to assess clinical efficacy.

**Results:**

The patients were followed up for 8–36 months with an average of 19.48 months. There was no significant difference in the operation time and intraoperative blood loss between the two groups (*P* > 0.05). In the control group, the Cobb angles of the cervical spine were 5.13° ± 1.28°, 10.46° ± 1.07°, and 9.72° ± 1.43° before and after the operation. The observation group was followed by the Cobb angle of the cervical spine before and after the operation. They were 4.96° ± 1.39°, 11.67° ± 0.93°, and 11.13° ± 1.19°, respectively; the JOA scores before the operation, 1 week after the operation, and at the last follow‐up were (8.07 ± 1.13) points and (13.57 ± 0.82) points, and (14.19 ± 0.96) points, respectively; the IR was 86.52% ± 9.33%. The preoperative, postoperative 1 week, and last follow‐up JOA scores in the observation group were (8.37 ± 1.29) points, (14.11 ± 0.93) points, and (14.95 ± 0.78) points respectively. The IR was 88.74% ± 8.16% in the scores; the VAS scores were (5.54 ± 0.89) points, (1.73 ± 0.71) points, and (1.48 ± 0.52) points in the preoperative, postoperative 1 week, and last follow‐up in the control group. The VAS scores were (5.81 ± 0.94) points, (1.82 ± 0.61) points, and (1.16 ± 0.49) points before, 1 week, and after the final follow‐up. The JOA score and IR, VAS score and preoperative comparison between the two groups were statistically significant (*P* < 0.05), but there was no statistically significant difference between the two groups (*P* > 0.05).

**Conclusion:**

The new open‐powered nail anterior cervical plate system can achieve the same clinical effect as the traditional anterior cervical plate fixation in anterior cervical surgery, but it can simplify the operation process, effectively make up for the shortcomings of the traditional anterior cervical plate operation, and obtain satisfactory clinical application effect, which is worthy of clinical promotion.

## Introduction

With social development, the incidence rates of cervix‐related diseases such as cervical spondylosis and traumatic fractures are gradually increasing due to changes in lifestyle and work methods; in turn, these ailments are gradually becoming common clinical issues. For such diseases, many patients with ineffective conservative treatment and severe spinal cord injury are currently treated by surgery. After a long period of clinical practice, anterior cervical surgery can be used to directly decompress anterior pressure of the spinal cord, while providing immediate stability to the cervical spine; this method improves cervical lordosis and is becoming a classic surgical treatment for cervical‐spine‐related diseases.

Since Robinson and Smith^1^ first used anterior cervical surgery to treat cervical spondylosis in the 1960s, after a long period of clinical practice, anterior cervical surgery has become a classic operation to treat cervical‐related diseases. This kind of operation can directly reduce the pressure in front of the spinal cord, such as the degenerative intervertebral disc, and at the same time provide strong fixation through cage or titanium mesh bone graft and anterior cervical internal fixation plate system, maintaining the stability of the cervical spine after operation and improving the physiological curvature of the cervical spine^2^, ^3^. Subsequently, various cervical anterior plate systems were developed with this technique, including: (i) bicortical screws (cortical bone screws) non‐locking non‐robust; (ii) single cortical screws (cancellous bone screws) locked sturdy; (iii) cortical screws (rotatable cancellous bone screws) angled half‐restricted; and (iv) single cortical screws (slip cancellous bone screws) slip semi‐limited^4^. However, the traditional cervical anterior plate has certain limitations in the design and operation of the steel plate itself. The traditional cervical anterior titanium plate will slip during the upper plate process, so that it is difficult to precisely control the entry point. This means that the titanium plate cannot achieve the expansion and compression effect required by the operation, which can prolong the operation time. When using the expansion screw hole as the screw entry hole of titanium plate, the expansion screw should be taken out, which will increase the amount of bleeding, make it difficult to expose, increase the difficulty of the operation, and increase the incidence of complications. Common complications such as spinal cord injury, nerve damage, vascular injury, lung injury, esophageal injury, cerebrospinal fluid leakage, formation of epidural hematoma, and failure of internal organs, often result in surgical failure^5^, ^6^, ^7^, ^8^.

In order to address the current technical problems in clinical operation by the cervical anterior nail plating system, this work provided a novel open‐dynamic cervical anterior nail plating system (the new open‐powered cervical spine system), which was applied in anterior cervical vertebral surgery to evaluate its clinical efficacy.

Through retrospective comparative analysis of the clinical data of 329 orthopaedic patients treated with cervical anterior decompression, cage or titanium mesh graft fusion, new opening dynamic nail plate or traditional cervical anterior screw plate, the purpose of this study is: (i) to design the new open‐powered nail plate system; (ii) to explore the advantages of surgical operation by using the new open‐powered nail plate system; and (iii) to evaluate the clinical efficacy of the new open‐powered nail plate system.

## Materials and Methods

### *General Information*

This was a retrospective analysis of 329 patients admitted to our hospital from January 2015 to June 2017 and treated with cervical anterior decompression, cage or titanium mesh graft fusion, new open‐powered dynamic nail plate or traditional cervical anterior screw plate fixation. The patients with cervical disease included 192 males and 137 females, aged 39–83 years, averaged 61.2 years. Among them, there were 74 cases of cervical fracture and dislocation or instability, 82 cases of cervical spondylotic myelopathy, 26 cases of cervical disc herniation, 138 cases of mixed cervical spondylosis, and nine cases of cervical spondylitis or brucellosis. The disease course ranged from 3 days to 17 years with an average of 38.67 months. All patients underwent cervical X‐ray, computed tomography (CT), and magnetic resonance imaging (MRI) examinations upon admission. The preoperative and postoperative follow‐up data of all patients were complete and clear. A total of 154 (control group) and 175 (observation group) cases had fixation with the conventional cervical‐ and new open‐powered dynamic nail plates, respectively. In the traditional cervical nail plate group, 119 cases were treated with anterior cervical discectomy and fusion (ACDF); with single‐segment involvement there were 8, 43, 27 and 18 in the C_3‐__4_, C_4‐__5_, C_5‐__6_ and C_6‐__7_ segments, respectively; and with two‐segment involvement there were 13 cases in the C_4‐__5_ and C_5‐__6_ segments, 10 cases in the C_5‐__6_ and C_6‐__7_ segments, and 35 cases using anterior cervical corpectomy decompression and fusion (ACCF). There were 136 ACDF cases in the new open dynamic nail plate; with single‐segment involvement there were 11, 41, 29 and 17 cases in the C_3‐__4_, C_4‐__5_, C_5‐__6_ and C_6‐__7_ segments, respectively; and with two‐segment involvement there were 20 cases with C_4‐__5_ and C_5‐__6_ segments, 18 cases with C_5‐__6_ and C_6‐__7_ segments, and 39 cases with ACCF.

### *Inclusion and Exclusion Criteria*

#### *Inclusion Criteria*

The inclusion criteria were as follows: (i) patients met the symptoms and signs of cervical spondylosis, cervical fracture, cervical disc herniation, cervical spondylitis or brucellosis, confirmed by imaging and neurophysiological examinations; (ii) patients treated with anterior cervical surgery using the conventional cervical‐ and new open‐powered dynamicnail plates; (iii) patients completed preoperative and postoperative imaging examinations and follow‐up; (iv) the main evaluation indicators included intraoperative blood loss, cervical vertebra Cobb angles, VAS scores, and the Japanese Orthopaedic Association (JOA) scores; and (v) retrospective comparative study.

#### *Exclusion Criteria*

The exclusion criteria were as follows: (i) patients with heart, brain, kidney, and other important organ diseases; (ii) cervical diseases caused by infection or tumor; and (iii) patients unable to cooperate with surgical treatment.

### *The Surgical Method*

With the patient in the cervical posterior position, the front of the right cervical row was incised, followed by incision of the skin, subcutaneous layers, platysma and superficial cervical fascia, bluntly separated in front of the target vertebral body. Then, the prevertebral fascia was cut to determine the lesion. After vertebral body clearance, C‐arm X‐ray fluoroscopy confirmed the segment, with a distractor used to expand the adjacent vertebral body of the surgical lesion intervertebral space; curettage of the intervertebral disc or vertebral body was performed by subtotal cut, with full decompression. The spinal cord then had no pressure, and the cartilage end plate was placed in a suitable size cage or length of titanium mesh. C‐arm perspective was used to determine whether the position of the cage or titanium mesh was accurate and good. The appropriate size of the new open‐powered nail plate or traditional cervical anterior screw plate close to the front edge of the vertebral body was selected and locked in place after a good positioning. After rinsing, a negative pressure drainage tube was placed after complete hemostasis; the layers were sawn and the surgical incision made. The drainage tube was indwelled for 1~3 days according to the drainage condition. Three days after the operation, proper function exercise was allowed and the patient was permitted to get out of bed.

### *Efficacy Evaluation*

A total of 329 patients were reviewed regularly, and routine cervical X‐ray and MRI examinations were performed to assess the stability, internal fixation position, and bone graft fusion of the cervical spine. The cervical Cobb angle, pain visual analogue scale (VAS) score, and the JOA spinal function score^9^ were recorded, and the neurological improvement rate (IR) was determined [IR = (final follow‐up score − preoperative score)/(17‐surgery Pre‐score) × 100%] to comprehensively evaluate the clinical efficacy of the new open‐dynamic nail plating system.

#### *Pain VAS score*

The VAS is a method of simply measuring pain intensity in clinical practice. The basic method is to use a swimming ruler with a length of about 10 cm, with 10 scales on one side, and “0” and “10” ends on both ends. The “0” mark means no pain, and the “10” mark means the most severe pain that could be endured^10^, ^11^. In clinical use, the scaled side is turned back to the patient, and the patient is marked on the ruler with a corresponding position that represents his or her pain level, and the physician evaluates the score based on the position indicated by the patient. The clinical evaluation is divided into “excellent” by “0‐2”, “good” by “3‐5”, “corable” by “6‐8”, and “poor” by “8”.

#### *JOA score system*

The modified JOA scoring system was used to evaluate the neurological status. The JOA score system includes four sections: four limbs motor dysfunction, four limbs sensory deficit, trunk sensory deficit, sphincter dysfunction. The maximum score of 17 indicates normal function. An IR was calculated as: IR = (final follow‐up score − pre‐operative score)/ × (17‐pre‐operative score) × 100%. The IR was then used to define the surgical outcome: excellent (IR≥75%), good (75% > IR≥50%), fair (50% > IR≥25%), and poor (IR < 25%).

### *Statistical Methods*

Analysis was performed with the SPSS 21.0 statistical software. Measurement data were expressed as mean ± standard deviation (SD). Paired *t*‐test was used before and after the operation. The control and observation groups were compared by paired *t*‐test. *P* < 0.05 was considered statistically significant.

## Results

There was no significant difference in gender, age, preoperative JOA scores, preoperative cervical vertebra Cobb angles, and preoperative VAS scores between the two groups (*P* > 0.05).

### *Surgery Information*

In the control group, operation time was 70–150 min, with an average of 83.4 ± 10.6 min; blood loss was 50–150 mL, averaging 68.2 ± 11.7 mL. The observation group showed an operation time of 60–120 min, averaging 72.8 ± 9.3 min; blood loss was 30–130 mL, averaging 56.3 ± 12.2 mL. Operation time and blood loss between the two groups were significantly different (*P* > 0.05). In the operation, the upper plate is fixed without removing the distraction nail, the titanium C‐shaped opening side is directly inserted into the special opening nail, and the nail is set in a stable and pressurized state, which not only ensures pressurization but also means it can be controlled accurately (Fig. 3).

### *Clinical Improvement*

In the control and observation groups, preoperative, postoperative 1 week, and last follow‐up cervical vertebra Cobb angles, JOA scores, and VAS scores are shown. The IR in the control group was 86.52% ± 9.33%, while the observation group showed 88.74% ± 8.16%. There were significant differences between the two groups in postoperative one‐week and final follow‐up cervical Cobb angle, JOA score, and VAS score (*P* < 0.05), but there were no statistical significant differences preoperatively between the two groups (*P* > 0.05).(Tables 1, 2, 3).

**Table 1:** Preoperative and postoperative Cobb angle in the control and observation groups ($\bar{x}\pm s$)

	Groups	Preoperative	One week after surgery	Last follow‐up
	Control group	5.13° ± 1.28°	10.46° ± 1.07°	9.72° ± 1.43°
	Observation group	4.96° ± 1.39°	11.67° ± 0.93°	11.13° ± 1.19°

*Note*: In the two groups, the improvement in Cobb angle before and after surgery (*P* < 0.05), and the improvement in Cobb angle between the two groups were compared (*P* > 0.05).

**Table 2:** Preoperative and postoperative JOA score in the control and observation groups ($\bar{x}\pm s$)

	Groups	Preoperative	One week after surgery	Last follow‐up
	Control group	8.07 ± 1.13	13.57 ± 0.82	14.19 ± 0.96
	Observation group	8.37 ± 1.29	14.11 ± 0.93	14.95 ± 0.78

*Note*: In the two groups, the improvement in JOA score before and after surgery (*P* < 0.05), and the improvement in JOA score between the two groups were compared (*P* > 0.05).

**Table 3:** Preoperative and postoperative VAS scores in the control and observation groups ($\bar{x}\pm s$)

	Groups	Preoperative	One week after surgery	Last follow‐up
	Control group	5.54 ± 0.89	1.73 ± 0.71	1.48 ± 0.52
	Observation group	5.81 ± 0.94	1.82 ± 0.61	1.16 ± 0.49

*Note*: In the two groups, the improvement in VAS scores before and after surgery (*P* < 0.05), and the improvement in VAS scores between the two groups were compared (*P* > 0.05).

### *Complications Information*

Postoperative drainage volume was less than 30 mL, and drainage tubes were removed. The second day after operation, the cervical collar was moved down. In the control group, there were 23 cases of hoarseness and 12 of dysphagia in the postoperative period; the observation group had 17 hoarseness and five dysphagia cases. They all began to be relieved on postoperative day two and returned to normal around January. The average follow‐up time was 19.48 months (8–36 months). No loosening, rupture, or prolapse of the plate and screws occurred in either groups. At the last follow‐up, all 329 patients had stable bone fusion.

**Figure 1:**
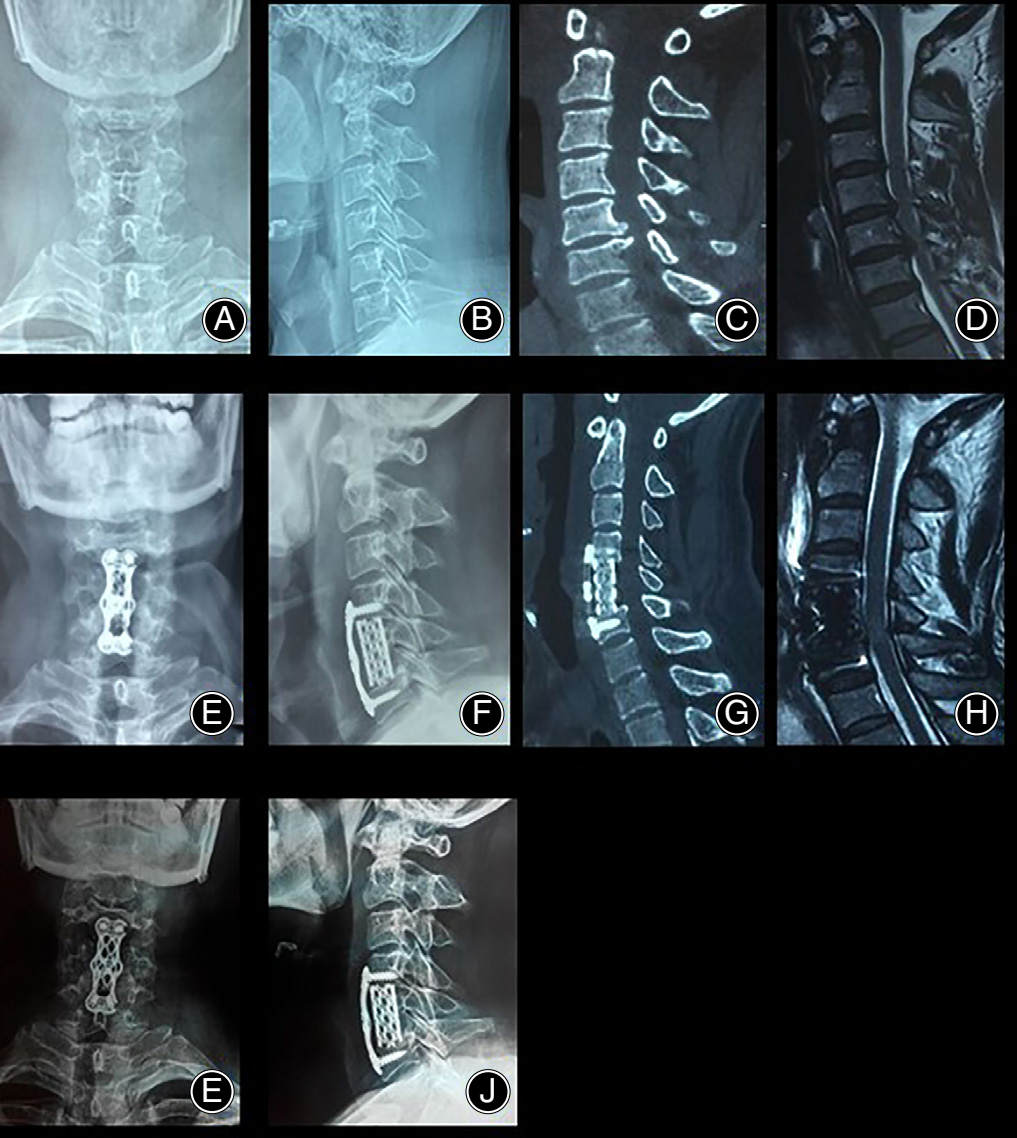
A representative case. Male patient, 56 years old, with numb hands and weakness in both lower limbs for 2 years, exacerbated by 1 month. (A and B) Preoperative x‐ray report of cervical curvature straightening. (C) CT report of osteophyte formation in the posterior margin of 5–6 segments of cervical spine. (D) Preoperative MRI showed that the anterior compression of 5–6 cervical spinal cord was obvious. (E and F) After ACCF operation, titanium mesh and titanium plate were fixed firmly. (G) Postoperative CT showed that the posterior edge of cervical vertebrae 5–6 had been removed. (H) Postoperative MRI showed that the compression of cervical spinal cord at 5–6 segments had been relieved, and there was no obvious compression of spinal cord. (I and J) There was no obvious subsidence of titanium mesh X‐rays 3 months after surgery.

## Discussion

### *Design of the New Open‐Powered Nail Plate System*

**Figure 2:**
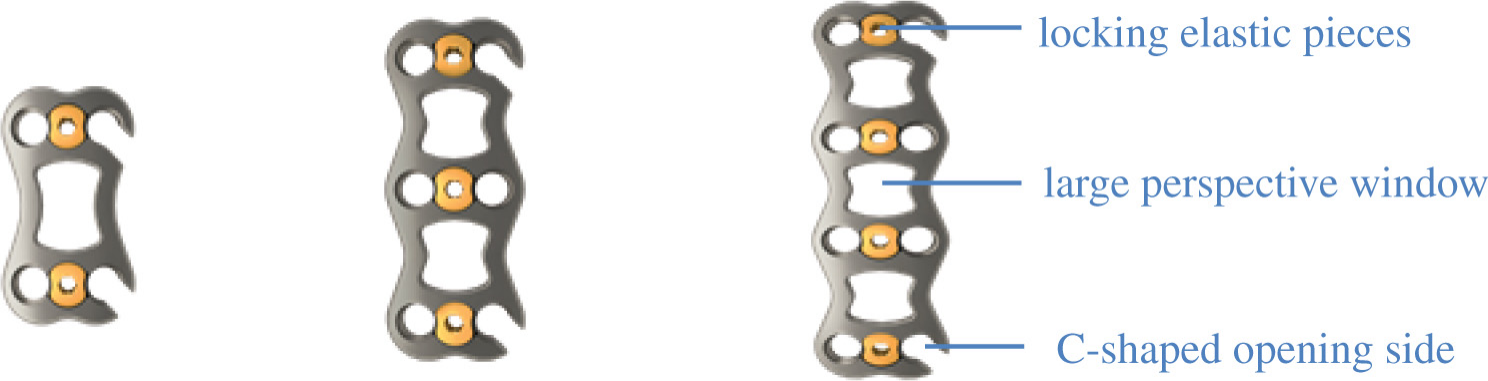
various models of new open‐powered nail plate system. The locking elastic pieces are arranged above the nail holes at both ends of the titanium plate to effectively prevent the loosening of screws; The large perspective window is facilitate observation and intraoperative bone grafting; The titanium C‐shaped opening side is directly inserted into the special opening nail, which not only ensures pressurization but also can be controlled accurately.

**Figure 3:**
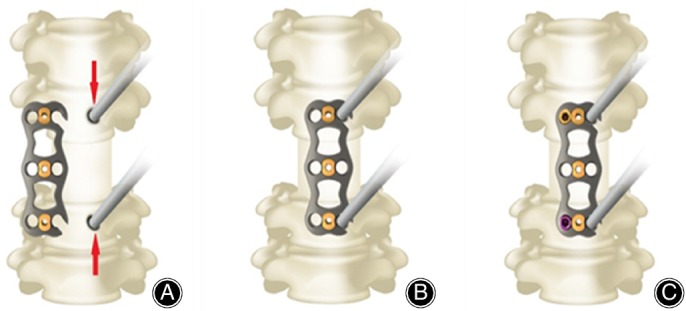
Schematic diagram of the surgical procedure for the new open‐powered nail anterior cervical plate system. (A) The distraction nail remain pressurized. (B) The upper plate is fixed without removing the distraction nail, the titanium C‐shaped opening side is directly inserted into the distraction nail. (C) Screw in the other two locking nail first, then remove the distraction nail, and finally the distraction nail hole is used as remaining locking nail fixing hole.

The new open‐powered nail plate is made of medical titanium alloy. Its components include steel plates, fixing screws, and a locking shrapnel. Steel plate width is 17 mm; lengths are 22–65 mm (with 3 mm increment) with 2.2 mm thickness. The screw length range is 10–20 mm, with a diameter of 4 mm. The locking elastic pieces are arranged above the nail holes at both ends of the titanium plate to effectively prevent the loosening of screws and increase safety. Both ends of the titanium plate were designed with a sloped edge and a smooth surface to reduce stimulation of the surrounding soft tissue. Meanwhile, the titanium plate had a pre‐curved design, which not only reduces the use of a plate bender, but also makes the titanium plate more conformable to the bone surface, effectively avoiding stress. It should be noted that the middle part of the titanium plate has a large perspective window to facilitate observation and intraoperative bone grafting. The screws were designed as self‐tapping screws, which can be divided into fixed and adjustable angles. Meanwhile, the colors can be distinguished. This not only reduces the use of wiretapping, but also quickly discerns the diameter and type (Figs 2 and 3).

### *Advantages of Surgical Operation by Using the New Open‐Powered Nail Plate System*

The nail holes at both ends of the titanium plate are “C” shaped and not completely closed. In clinical practice, we observed that for patients with cervical fractures and dislocations, after anterior decompression surgery, the open nail in front of the upper plate should be removed after traditional cervical titanium plate fixation. The nail hole is used as the screw fixing hole for the fixed titanium plate. At this time, the original opening nail hole will cause large amounts of bleeding, affecting the visual field of operation. Meanwhile, because the titanium plate is not fixed, it slides in front of the vertebral body and elevates the upper plate to set the nail. The most important challenge is due to the removal of nails; at this time, the implantation of the cage or titanium mesh cannot be due to pressure fixation. The new open‐powered nail plate system could well avoid these problems. In the process, the upper plate is fixed without removing the distraction nail, the titanium C‐shaped opening side is directly inserted into the special distraction nail, and the nail is set in a stable and pressurized state, which not only ensures pressurization but can also be controlled accurately. In this simple and accurate process, reduce nail‐hole bleeding and decrease operation time.

### *Efficacy of the New Open‐Powered Nail Plate System*

Through patient follow‐up in the control and observation groups, we found that although there were no significant differences in operation time and intraoperative blood loss between the two groups, operation time and blood loss in the observation group were significantly lower. In the control group, it was demonstrated that application of the new open dynamic nail plating system in anterior cervical spine surgery could optimize the surgical operation, simplify the upper plate process, and reduce operation time. There were significant differences in Cobb angle, JOA score and IR, and VAS score preoperatively; however, after the first week and last follow‐up, there were no significant differences between the two groups. The results show that the new open‐powered nail plate system could effectively maintain the stability of the cervical spine, effectively restore the physiological curvature of the cervical spine, significantly improve the symptoms of patients, and obtain similar clinical effects with the traditional cervical plate.

### *Indications and Deficiencies of the New Open‐Powered Nail Plate System*

The indications of the new open‐powered nail plate system include the following: (i) cervical degenerative disease and obvious symptoms, with ineffective conservative treatment, e.g. cervical spondylosis and cervical posterior longitudinal ligament ossification; (ii) acute and chronic cervical trauma, such as cervical fracture and dislocation, non‐fracture‐dislocation, cervical spinal cord injury, and instability of old cervical spine injury; (iii) cervical benign and malignant tumors; (iv) cervical infections, such as cervical tuberculosis and Brucella infection in the cervical spine; and (v) congenital cervical disease and metabolic diseases such as ankylosing spondylitis. Although the new open dynamic nail plating system has a wide range of indications and satisfactory clinical outcomes, long‐term follow‐up of patients is required for further assessment.

In conclusion, the new open‐powered nail plate system for anterior cervical spine surgery has a satisfactory curative effect and provides an effective stabilizing effect on the cervical spine. Meanwhile, it simplifies the surgical procedure and shortens operation time. It is safe and effective, and therefore worthy of clinical promotion.
